# Dogs’ ability to follow temporarily invisible moving objects: the ability to track and expect is shaped by experience

**DOI:** 10.1007/s10071-022-01695-5

**Published:** 2022-09-27

**Authors:** Miina Lõoke, Orsolya Kanizsar, Cécile Guérineau, Paolo Mongillo, Lieta Marinelli

**Affiliations:** Department of Comparative Biomedicine and Food Science, University of Padua, Viale dell’Università 16, 35020 Legnaro, PD Italy

**Keywords:** Dog, Expectancy violation, Motion perception, Occlusion, Prediction, Visual tracking

## Abstract

Visually tracking a moving object, even if it becomes temporarily invisible, is an important skill for animals living in complex environments. However, this ability has not been widely explored in dogs. To address this gap of knowledge and understand how experience contributes to such ability, we conducted two experiments using a violation of expectation paradigm. Dogs were shown an animation of a ball moving horizontally across a screen, passing behind an occluder, and reappearing with a timing that was faster, slower or congruent with its initial speed. In the first experiment, dogs (*N* = 15) were exposed to the incongruent conditions without prior experience; while in the second experiment, dogs (*N* = 37) were preliminarily exposed to the congruent stimulus. Dogs of the first experiment did not exhibit a surprise effect, as measured by latency to look away from the expected stimulus presentation area, in response to the incongruent conditions, suggesting they had not formed an expectation about the timing of reappearance. However, their latency to orient towards the reappearing ball depended on the condition, suggesting they were able, to some extent, to visually keep track of the stimulus’ trajectory. Dogs of the second experiment were surprised when the ball stayed behind the occluder longer than expected, but showed no difference in latency to orient across conditions. This suggests they had overcome the visual tracking mechanism and had formed expectations about the timing of reappearance. In conclusion, dogs seem to use a low-level mechanism to keep visual track of a temporarily disappearing moving object, but experience is required to make expectation about its trajectory.

**Supplementary Information:**

The online version contains supplementary material available at 10.1007/s10071-022-01695-5.

## Introduction

Many animals live in complex environments, where visually scanning the surroundings and tracking moving objects is essential for several aspects of life, such as escaping from predators, catching prey, or mating. However, objects in motion might not always stay in the full view of the observer, as they become temporarily invisible, when passing behind other obstacles in the surrounding. For instance, a prey running in a forest might get hidden by vegetation, remaining invisible for some time before reappearing at a different place. The predator needs to correctly predict the prey’s reappearance; otherwise, tracking of it will be ineffective.

Several studies have looked into animal’s ability to maintain the representation of objects disappearing from the observer’s view, and in particular, the topic has been thoroughly researched in birds (Chiandetti and Vallortigara 2011; Fontanari et al. 2011; Regolin et al. 1995; Vallortigara et al. 1998). However, the ability to use information about the object’s movement, for instance to track its position even when it disappears from view, or to predict where/when it should reappear, received less attention. Human infants, at around six months of age, are not able to predict the reappearance of a moving object at the first presentation, as they orient towards the location of disappearance or the central area of the occluder, instead of orienting towards the location of reappearance (Nelson 1971; Von Hofsten et al. 2000). Nevertheless, their performance improves quickly within the first three trials and thereafter remains constant over the following presentations (Von Hofsten et al. 2000). If the stimulus stays behind an occluder for longer than expected, the infants show distress until the reappearance attracts their attention again (Meicler and Gratch 1980). The ability to predict an object reappearance has also been described in non-human animals: for instance, keas have been shown to simultaneously remember the identity of two objects moving behind an occluder and predict the reappearance of the preferred object (Bastos and Taylor 2019). Furthermore, Churchland and colleagues (2003) recorded eye movements of rhesus monkeys watching a moving stimulus and found that eyes kept moving even when the object temporarily disappeared, although with decreased speed. This owes to a mechanic, low-level mechanism, which has a clear functional significance in allowing to keep orientation towards a moving object even across short gaps in visibility. At the same time, the slowing down of eye movement was remarkable when the disappearance was unexpected (e.g., a sudden, short blink), but less so when it was predictable (e.g., the object passing behind an occluder). This result highlights how the limited accuracy of the low-level mechanism can be improved through the cognitive appraisal of the physical context. The study also shows that repeated exposures to the stimulus, even if just to the blinking one, allow monkeys to overcome the slowing down, and actually shift their gaze to where the stimulus would eventually reappear, with anticipate timing. Thus, although the ability to maintain a representation of an occluded object is present from early age and without experience (Bastos and Taylor 2019; Freire and Nicol 1999; Regolin et al. 2005; Vallortigara et al. 1998), direct experience represents an important contribution to the ability to track and predict the trajectory of a temporarily occluded moving object.

In recent decades, dogs have gained popularity as a model in comparative cognition research, with most of the studies using visual stimuli (Bensky et al. 2013). However, the knowledge about dogs´ visual perception is far from being comprehensive. Particularly little attention has been paid to dogs’ perception and elaboration of motion information, in spite of suggestions that motion perception is a critical aspect of dogs’ vison (Miller and Murphy 1995). Only recently some studies have started to look into this topic, exploring some basic sensory features of dogs’ motion perception, such as the detection of coherent motion (Kanizsár et al. 2017, 2018) and the minimum detectable velocity (Lõoke et al. 2020). Other researchers have focused on dogs’ perception of biological motion, suggesting that dogs are sensitive to it (Delanoeije et al. 2020; Eatherington et al. 2019; Kovács et al. 2016), although with peculiarities with regards to which features are relevant in determining dogs’ attention to biological motion (Eatherington et al. 2021). Two recent studies have also looked into dogs´ ability to track moving objects (Völter et al. 2020; Völter and Huber 2021a). In particular, Völter and Huber (2021a) found that dogs followed closely a rolling object and made predictions based on contact causality. Similarly, Völter and colleagues (2020) showed that, when presented with a video of two players throwing a frisbee back and forth, dogs can visually track the frisbee with a high accuracy. Moreover, with increasing experience, their motion tracking turned into an anticipatory looking behavior, as dogs turned their gaze to the catcher before the frisbee arrived. Much as these studies provided an important insight in dogs’ motion tracking abilities, it does not shed light on dogs’ ability to predict the spatiotemporal trajectory of an object that disappears from the visual scene for a limited time.

Several studies have used the violation of expectation paradigm in dogs to explore sensitivity to certain phenomena. The paradigm builds on the idea that exposure to an inconsistent sequence of two events involving the phenomenon under study should lead to a surprised reaction (Winters et al. 2015). In dogs, surprise is often operationalized as a longer time spent looking at the inconsistent pairing, compared to the consistent one (Adachi et al. 2007; Mongillo et al. 2021; Pattison et al. 2010, 2013; West and Young 2002; Zentall and Pattison 2016). The methodology has been applied to several aspects of dogs’ cognition and perception, including numerical competence (West and Young 2002), recognition of conspecifics (Mongillo et al. 2021) and humans (Adachi et al. 2007), size and color consistency (Pattison et al. 2013) and object permanence (Pattison et al. 2010; Zentall and Pattison 2016). The same paradigm has also been applied in studies using moving and disappearing objects, although not aimed at investigating dogs’ ability to process motion information per se. For instance, Müller and colleagues (2011) found that female dogs show the surprise effect after being exposed to a ball disappearing behind a barrier and having a different size at reappearance. Furthermore, Völter and Huber (2021b) found a surprise effect in response to a ball disappearing behind a barrier that was too thin to occlude the ball. Therefore, the violation of expectation paradigm seems a proper methodology to assess dogs’ ability to predict the reappearance of a moving object possibly eliciting a surprise effect when the reappearance of the moving stimulus is incongruent with dog’s expectation.

The aim of the current study was to assess dogs’ ability to predict the time of reappearance of a moving object that had disappeared behind an occluder. To reach this aim, dogs were shown animations of a ball moving horizontally at a constant speed passing under an occlude; whereas, the time spent behind it was varied, being shorter, longer or coherent with the ball’s initial speed. We hypothesized that, if dogs are able to keep track of the spatiotemporal trajectory of the ball even when occluded, they would orient to the location of reappearance at the correct timing—hence, we would observe a delayed orienting response in the fast condition and an anticipated orienting response in the slow condition, compared to the congruent one. Moreover, if dogs are able to form expectations about the correct timing of reappearance we would observe a surprised reaction if the time spent by the ball behind the occluder did not correspond to the one expected. Finally, to clarify the role of experience in shaping the ability to track and predict spatiotemporal trajectories, the present study included two experiments: in the first, dogs were presented with the stimuli without having any prior experience of them, while in the second experiment dogs were preliminary exposed to the coherent stimulus prior to being presented with the incongruent ones. If indeed experience is crucial in shaping the ability, we should expect a different pattern of results between the two experiments; conversely, if dogs are spontaneously able to use characteristics of motion, no difference should be found between the two experiments.

## Methods

### Experiment 1

#### Subjects

The sample consisted of 15 companion dogs, out of which 8 were males and 7 were females, dogs’ average age was 3.5 ± 1.0 years. Five dogs were mixed breeds and the remaining were from various breeds (detailed demographic information is presented in Table S1). Dogs were recruited through the database of volunteers at the Laboratory of Applied Ethology of the University of Padua. The criteria for recruitment were that dogs were in good health and comfortable in a laboratory environment.

#### Experimental setting

**Fig. 1:**
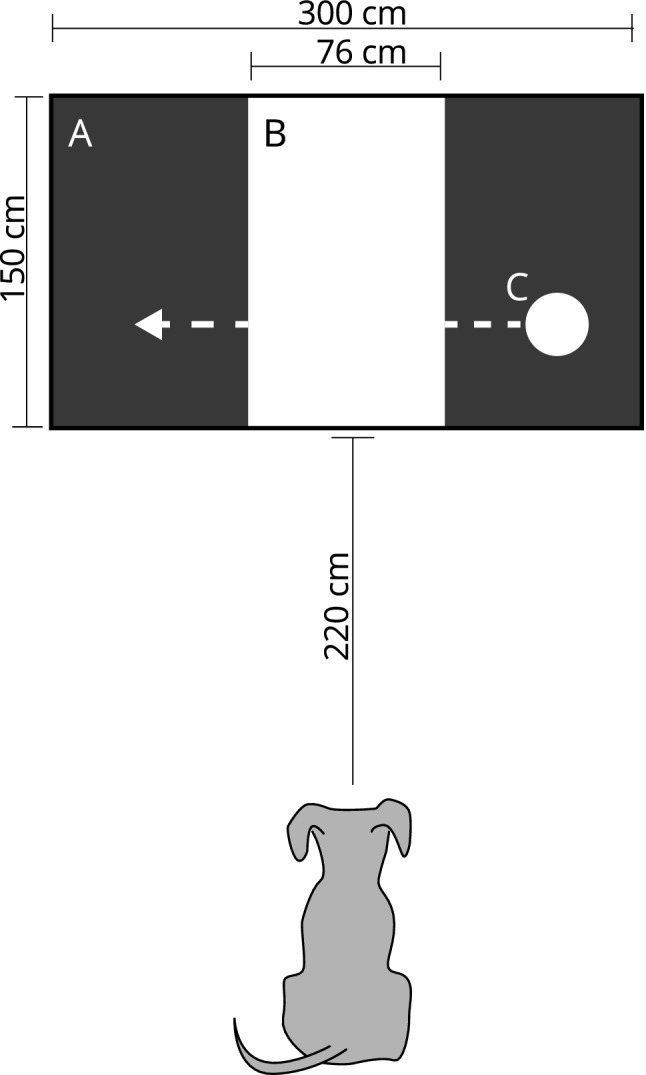
A schematic view of the experimental setting, illustrating the position and size of the projection area (**A**) and the plastic panel (**B**), the distance of the dog from the presentation area and one of the possible locations of the moving stimulus (**C**)

The experiment was conducted in a dimly lit quiet room, measuring 4.7 × 5.8 m. The stimuli were presented on a white wall using a video projector (Epson MG850 HD, Epson Corporation, Suwa, Japan). The projection area was 300 cm wide. A white plastic panel (width 76 cm and height 150 cm) was placed at the center of the projection area, leaving 112 cm free on both sides (Fig. 1). During the trials, dogs faced the projection area at a distance of 220 cm. Trial presentation was controlled by an experimenter who sat at the back of the room, using a Dell laptop (Dell, TX, USA). Two loudspeakers (Hercules XPS 2.0 60, Hercules Computer Technology, CA, USA) connected to the laptop, were placed on the floor on both sides of the screen. To record the trials, a Canon XA20 (Canon, Tokyo, Japan) camcorder, set on infrared mode, was placed at floor level between the dog and the screen, facing the dog’s head from a distance of 150 cm. A second camera was mounted on the ceiling above the dog and facing down towards the dog.

#### Stimuli

The stimuli consisted of computer-generated animation representing a circle of 40 cm of diameter (hereafter referred to as ball), filled in orange on a black background. The ball entered the projection area from either side, with its center at 60 cm from ground level, and crossed horizontally the entire area with a constant speed of 0.5 m/s, before disappearing on the opposite side. In the middle of the projection area, the ball disappeared temporarily behind a white rectangle, projected onto the plastic panel. The plastic panel had the function of making the disappearance of the ball more realistic. Three animations (hereafter: conditions) were used in the experiments, differing in how long the ball remained invisible, measured from the first to the last frame in which the ball was fully hidden by the panel:

- the congruent condition (Video S1), where the ball remained invisible for 0.72 s, corresponding to the time needed by the ball to cross the barrier, had it maintained the constant speed of 0.5 m/s;
- the slow condition (Video S2), where the ball remained invisible for 1.7 s, corresponding to the time needed by the ball to cross the barrier, if it slowed down to 0.2 m/s constant speed when behind the panel;
- the fast condition (Video S3), where the ball remained invisible for 0.2 s, corresponding to the time needed by the ball to cross the barrier, if its constant speed was 1.8 m/s when behind the panel.

Each animation started with an attention grabber, which was presented on the same side of the screen from where the ball eventually appeared. The attention grabber was a white figure shaped as a pin, similar to the size of the ball, oscillating around its center and accompanied by a frequency modulated harmonic sound if needed (see details of the procedure below). All animations were created with Adobe Flash (Adobe Systems, Mountain View, California, USA) and presented using Flash Player (Adobe Systems, Mountain View, California, USA).

### General experimental procedure

The presentation of animations occurred in sessions composed of three trials. The dogs were held gently by the owners sitting behind them without interfering with dogs’ behavior. The owners were unaware of the aim of the experiment and were instructed to look at their own lap during the presentations, so not to influence the dogs’ behavior. Once the dog was positioned correctly, the experimenter started the presentation, showing the attention grabber. If the dog did not orient towards the attention grabber spontaneously, its attention was captured by quickly moving a laser pointer over the presentation area; if this did not capture the dogs´ attention, the accompanying sound was turned on. As soon as the dog oriented towards the attention grabber, the experimenter started the actual ball presentation. After the presentation had finished (i.e., the ball had disappeared on the opposite side of that of entrance), the experimenter waited until the dog shifted the attention away from the screen spontaneously, which marked the end of the trial. The owner was instructed to remain silent and motionless during and after the presentation, until being told otherwise by the experimenter. All three trials of a session were presented consecutively, without the dog leaving the testing room. The average time between the trials was between one to two minutes.

### Experimental design

**Fig. 2:**
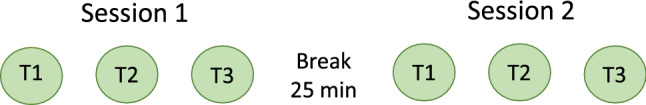
The experimental design of Experiment 1. Green circles with T represent test trials where one of the three experimental conditions was presented

Dogs underwent two sessions of three trials each representing one of three different conditions (Fig. 2). The order by which the three conditions were presented within each session was randomized and counterbalanced within the group of dogs. The entry side was the same across all trials for any given dog and counterbalanced within the group. The second session was carried out in the same day as the first, with a 25 min break between the two sessions.

### Data collection and analyses

Data regarding the dogs’ visual orientation was collected from videos with the Observer XT software (version 12.5, Noldus, Groeningen, The Netherlands). The data were collected with a continuous sampling method, from the moment the stimulus became visible until the dog spontaneously looked away from the screen after the stimulus had disappeared. Dogs’ visual orientation was coded as left or right, when the dog was oriented towards the part of the presentation area to the left or to the right of the plastic panel, as middle, when the dog was oriented centrally towards the plastic panel and elsewhere, if the dog was looking anywhere else in the room. Inter-observer reliability was assessed using data collected by a second observer, on a random subset of 30% of videos. Both observers were blind to the experimental condition since the projection area on the video were masked during their coding. The data collected by the two observers were highly correlated (Pearson’s correlation; looking left: *r* = 0.93, looking right: *r* = 0.98, looking middle: *r* = 0.98, looking elsewhere: *r* = 0.95). Only the trials in which the dogs were oriented towards the projection area for the entire time until the ball reached the panel and at least 40% of time after the balls’ reappearance from behind the panel were considered for further analyses. The 40% criterion was based on visual inspection of the data, which indicated such value as a relevant threshold—for dogs either looked for ≥ 40%, or for much less than that.

Data obtained were used to compute two variables. The variable “latency to reorient” indicated the time from the reappearance of the ball until the dogs oriented to the reappearance side. The value was negative if the dog was already oriented to such area before the ball reappeared. The variable was computed to assess whether dogs were looking at the area of reappearance consistently with the ball’s initial speed.

The second variable, “latency to look away”, indicated the time from the final disappearance of the stimulus until the dog shifted its’ orientation away from the presentation area. The variable was computed to be indicative of a possible surprise effect, hence of a violated expectation, induced by the incongruent timing of reappearance of the ball.

The actual order by which each dog was exposed to the presentations was determined after eliminating the presentations where the dogs did not pay sufficient attention, according to the criterion reported above for the exclusion of trials from the analysis. For example, if the dog only paid the required attention in the last two trials, then those trials were reclassified as the first and the second presentation and the previous trials were not considered for analysis. Since the overall number of usable presentations decreased across order number (i.e., overall fewer 6th trials were usable, than 5th trials and so on), to the aims of statistical analysis, the presentation order was reclassified as a three-level categorical variable (presentation order level). Level 1 included trials presented as 1st, level 2 included trials presented as 2nd or 3rd, and level 3 included trials presented as 4th, 5th or 6th.

To assess if the condition or the presentation order level affected the dogs’ timing to orient towards the area of reappearance of the ball, we fitted a generalized estimating equations model (GEE), where the dependant variable was the latency to reorient. The subject ID was included as random effect and the presentation order level and condition as random slopes within subject ID. The fixed factors were the presentation order level, the condition and their interaction. The dog’s age was included in the model as a covariate. A backward elimination procedure was used to obtain the final model. If a significant effect was found for any of the factors or the interaction, post hoc pairwise comparisons were run, with Sequential Bonferroni corrections for multiple comparisons. Moreover, analysis of the confidence intervals of the estimated mean in any condition and presentation order level was performed, to assess whether the latency to reorient was lower, higher or not significantly different from 0. The rationale for such analysis was to determine whether dogs were orienting to the area of reappearance with a timing that was coherent, anticipated or delayed compared to the actual reappearance.

A second GEE model was fitted to assess if dogs were surprised by the incongruent timing of reappearance. The dependent variable was the latency to look away from the presentation area after the end of the animation. The subject ID was included as random effect and the presentation order level and condition as random slopes within subject ID. The fixed factors were the presentation order level, the condition and their interaction. The dog’s age was included in the model as a covariate. To reach the final model, we performed backward elimination procedure. Post hoc analysis were conducted to assess differences between conditions and Sequential Bonferroni corrections were applied to post hoc pairwise comparisons.

All statistical analyses were performed with SPSS (SPSS ver. 26, IBM Inc., Armonk, NY, USA).

### Results

Thirty-eight trials were used for analysis, out of a theoretical potential maximum of 90. Out of the 38 presentations, 13 were of the congruent condition, 8 of the slow condition and 17 of the fast condition. Ten were the first presentations, 17 were either the second or the third presentations and 11 were one of the last three presentations. A median of 3 trials per subject were used (min = 1, max = 5, mean ± SD = 2.9 ± 1.5).

**Fig. 3:**
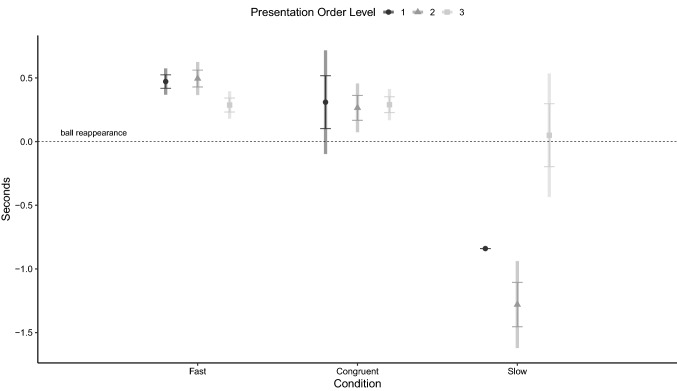
Estimated marginal means of the latency to reorient to the area after the panel, relative to the moment of reappearance of the ball (dashed line), in the Fast, Congruent and Slow conditions, when presented in different order levels (circle = level 1, triangle = level 2, square = level 3)*.* Error bars represent standard error of the estimate and rectangular areas represent 95% confidence intervals. Generalized Estimating Equation Model

The latency to reorient to the side of reappearance was significantly affected by the interaction between condition and presentation order level (Table 1). Estimated marginal means ± SE of the variable, for the three conditions and across trial order, are presented in Fig. 3. Confidence intervals for the estimates indicate that latency was not different from 0 when the congruent condition was presented as 1st trial, but was higher than 0 if the condition was presented as 2nd and 3rd trial (Level 2 of presentation order level) or in the following three trials (level 3 of presentation order level). In the fast condition, latency was always significantly higher than 0. In the slow condition, it was significantly lower than 0 when the condition was presented as 1st or 2nd and 3rd trial. Post hoc comparisons showed that the latency to reorient was lower in the slow condition than in either the congruent or fast condition, when the presentation order level was 1st (slow–congruent = − 1.14 ± 0.21, 95% CI = − 1.79 − 0.50, *p* < 0.001; slow–fast: = − 1.31 ± 0.05, CI = − 1.48 − 1.15, *p* < 0.001) or 2nd and 3rd (slow–congruent: = − 1.54 ± 0.19, 95% CI = − 2.16 − 0.92, *p* < 0.001; slow–fast: = − 1.77 ± 0.17, 95% CI = − 2.33 − 1.21, *p* < 0.001).

**Table 1:** Results of generalized estimating equations model assessing the effect of presentation order level and condition on the latency of the unexperienced dogs to reorient to the ball’s reappearance side

	Wald *χ*^2^	*df*	*p* value
Condition	73.074	2	< 0.001
Presentation order level	15.064	2	< 0.001
Age	0.512	1	0.474
Condition*presentation order level	29.324	4	< 0.001

**Fig. 4:**
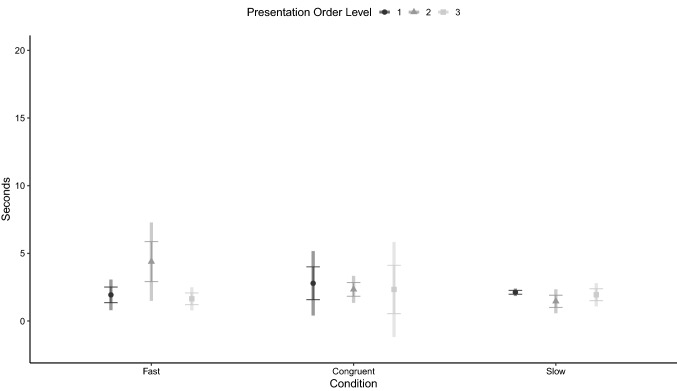
Estimated marginal mean of the latency to look away, in the Fast, Congruent and Slow conditions, when presented in different order levels (circle = level 1, triangle = level 2, square = level 3)*.* Error bars represent standard error of the estimate and rectangular areas represent 95% confidence intervals. Generalized Estimating Equation Model

The results of the GEE model assessing the dogs’ latency to look away from presentation area after the end of the animation are reported in Table 2, which shows that the variable was not affected by neither condition nor presentation order level. Estimated marginal means ± SE of the variable for the three conditions and across trial order are presented in Fig. 4.

**Table 2:** Results of the generalized estimating equations model assessing the effect of presentation order level and condition on dogs’ latency to look away from the presentation area after the final disappearance of the stimulus

	Wald *χ*^2^	*df*	*p* value
Condition	2.061	2	0.357
Presentation order level	2.027	2	0.363
Age	0.764	1	0.382
Condition*presentation order level	8.062	4	0.089

### Discussion

This first experiment assessed whether naïve dogs are able to predict the timing by which an object, moving with a constant speed and direction, would reappear after transiting behind an occluder.

The procedure involved exposing dogs to presentations in which the timing of object’s reappearance was faster, slower or congruent with its initial speed. Only in the slow condition, dogs were already oriented at the location of the ball’s reappearance, before the event happened. Conversely, in the congruent and fast conditions, they oriented at such location at the time of reappearance or, more often, later. The latter result could be explained by the fact that dogs’ attention was captured by the reappearing stimulus, while they were still oriented towards the barrier. This, however, would not explain why dogs were already oriented to the area or reappearance in the slow condition. A possibility for explaining this result is that dogs had formed an expectation about the spatiotemporal trajectory of the ball, based on its motion before disappearance. However, the lack of surprise in response to the incongruent timing of reappearance (more about this is discussed below) stands against such explanation. An alternative possibility is that dogs were resorting to visual tracking, a low-level perceptual/behavioral mechanism which allows an animal to maintain visual orientation towards a moving object (Land 1992, 2019; Scholl and Pylyshyn 1999). During tracking, gaze moves in accordance with the targets´ direction and speed and such motion can be maintained for a short period, even if the target is temporarily invisible (Churchland et al. 2003), providing the ability to keep track of objects through small spatiotemporal gaps (Scholl and Pylyshyn 1999). This mechanism nicely fits with dogs being already oriented to the area of reappearance in the slow condition and not in the fast condition. One may argue that a latency of zero should have been observed in the congruent condition, if dogs kept moving their gaze with the same speed it had when the ball disappeared. However, in visual tracking mode, gaze speed decreases as soon as the object disappears, as shown in both rhesus monkeys and humans (Churchland et al. 2003; Mrotek and Soechting 2007). Therefore, resorting to such a mechanism would not have allowed dogs to be already oriented to the area of reappearance in both the congruent and the fast condition, explaining the relatively long latency observed in these conditions.

Finally, no difference was found between the conditions in time spent looking at the presentation area after final disappearance of the stimuli, which suggests that dogs were unsurprised by the inconsistency in the timing of reappearance. In accordance with the violation of expectancy paradigm, it indicates dogs had not formed an expectation about the timing of reappearance. Since one possibility to explain the inability to form such expectation, is that dogs lacked specific experience with the stimuli, we conducted a second experiment, where dogs were given preliminary exposure to the congruent stimuli before viewing those with the incongruent timing.

## Experiment 2

### Methods

#### Subjects

The sample consisted of 37 naïve companion dogs, 18 dogs were males, and the remaining were females. The average age was 5.1 ± 2.9 years, 17 dogs were mixed breeds and the remaining from various breeds (Table S2). As in the previous experiment, dogs were recruited through the database of volunteers at the Laboratory of Applied Ethology of the University of Padua. The criteria for recruitment were that dogs were in good health and comfortable in a laboratory environment.

#### Experimental design

**Fig. 5:**
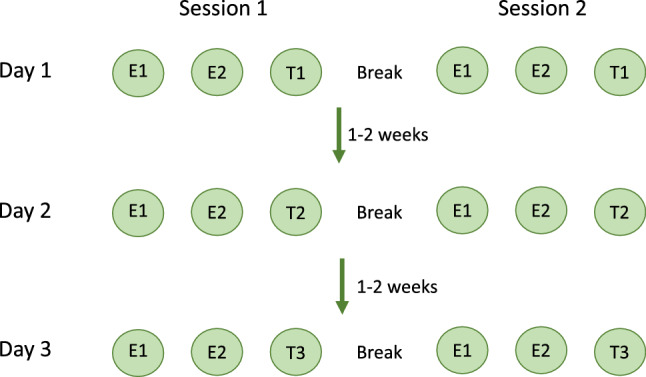
The experimental design of Experiment 2. Green circles represent trials, T represents test trials where one of the three experimental conditions was presented, and E represents experience trials where the congruent condition was projected

The experimental setting, stimuli and general trial procedure were identical to the ones of Experiment 1. However, the dogs in Experiment 2 underwent three testing days, each composed of two sessions of three trials (Fig. 5). Each session started with two experience trials, in which the congruent condition was presented; these were intended to provide the dogs with experience of the ball movement at a constant speed across the projection area. The third trial of the session was a test trial, in which one of the three conditions was presented. The same condition was presented in the test trials of the two sessions of any given day, and different conditions were presented in different days, so that each dog was eventually exposed to all three conditions twice. The order by which the conditions were presented across the three testing days was randomized and counterbalanced across the sample. The entry side was the same across all trials for any given dog and counterbalanced within the sample. The two sessions of the same day had a break of 25 min in between and the time interval between two testing days ranged from 1 to 2 weeks.

### Data collection and analyses

Data regarding dogs’ orientation were collected from videos obtained during the test trials. Data collection and trials selection were identical to the previous experiment. The data collected by the second observer were highly correlated (Pearson’s correlation; looking left: *r* = 0.89, looking right: *r* = 0.94, looking middle: *r* = 0.90, looking elsewhere: *r* = 0.93). Data analyses were identical to the previous experiment. In addition, a further model was run including data from both experienced and unexperienced dogs (i.e., those who took part to Experiment 1), and using as dependent variable the duration of the orientation towards the ball after its reappearance in the congruent condition, the dog’s name as random factor, and the group (unexperienced or experienced) as a fixed factor. The rationale for this analysis was to assess whether dogs of the experienced group had habituated to the congruent condition after the preliminary exposures.

### Results

Fifty-five test trials were used for analysis, out of a theoretical potential maximum of 222. Out of these, 19 were of the congruent condition, 23 were of the fast condition and 13 were of the slow condition. Twenty were presented as first, 22 were either the second (*N* = 13) or the third presentations (*N* = 9) and 13 were among the last three presentations (*N* = 4, 3 and 6 for the 4th, 5th and 6th presentation, respectively). A median of 2 test trials per subject were used (min = 1, max = 6, mean ± SD = 2.3 ± 1.1).

The relative duration of dogs’ orientation towards the ball after its reappearance in the congruent condition was 1.27 ± 0.27 s for the unexperienced dogs, and 1.37 ± 0.29 s for the experienced dogs. The GEE model revealed no significant effect of the group (Wald *χ*^2^ = 0.008, *p* = 0.928), nor of the order of trials in the experienced group (Wald *χ*^2^ = 2.04, *p* = 0.359) indicating that no habituation to the congruent condition occurred in the latter.

**Fig. 6:**
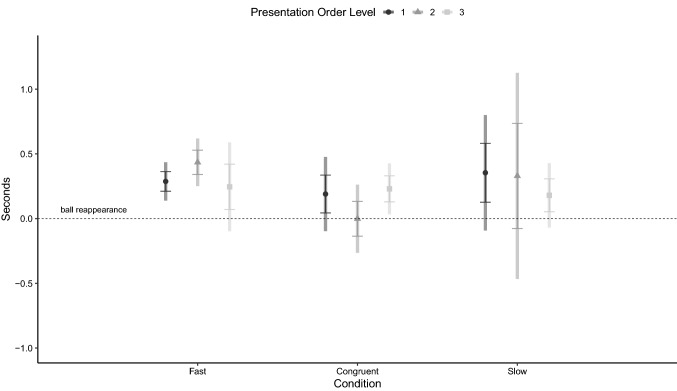
Estimated marginal means of the latency to reorient to the area after the panel, relative to the moment of reappearance of the ball (dashed line), in the Fast, Congruent and Slow conditions, when presented in different order levels (circle = level 1, triangle = level 2, square = level 3)*.* Error bars represent standard error of the estimate and rectangular areas represent 95% confidence intervals. Generalized Estimating Equation Model

The results of the GEE model assessing the dogs’ latency to reorient to the side of reappearance are reported in Table 3. The variable was not affected by any of the model terms (Fig. 6).

**Table 3:** The generalized estimating equations model assessing the effect of presentation order level and condition on latency of the dog to reorient to the ball’s reappearance side

	Wald *χ*^2^	*df*	*p* value
Condition	2.863	2	0.239
Presentation order level	0.326	2	0.850
Age	0.568	1	0.451
Condition*presentation order level	6.512	4	0.164

**Fig. 7:**
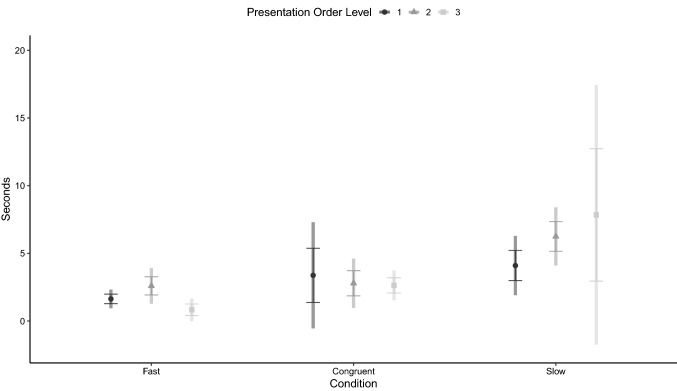
Estimated marginal mean of the latency to look away, in the Fast, Congruent and Slow conditions, when presented in different order levels (circle = level 1, triangle = level 2, square = level 3)*.* Error bars represent standard error of the estimate and rectangular areas represent 95% confidence intervals. Generalized Estimating Equation Model

The results of the GEE model assessing the dogs’ looking at the presentation area after final disappearance of the ball are reported in Table 4, which shows a main effect of condition. Estimated marginal means ± SE of the variable for the three conditions and across trial order are presented in Fig. 7. Dogs looked longer in the slow than in the congruent (mean difference ± SE = 3.13 ± 1.38 s, 95% CI = 0.03–6.23, *p* = 0.047) and in the fast condition (4.38 ± 1.76 s, 95% CI = 0.15–8.60, *p* = 0.039), while no significant difference was found between the latter two (*p* = 0.239).

**Table 4:** The generalized estimating equations model assessing the effect of presentation order level and condition on latency to look away from the presentation area after the final disappearance of the stimulus

	Wald *χ*^2^	*df*	*p* value
Condition	6.698	2	0.035
Presentation order level	1.656	2	0.437
Age	0.550	1	0.458
Condition*presentation order level	7.796	4	0.099

### Discussion

Contrary to the previous experiment, the time dogs remained oriented toward the presentation area after the final disappearance of the stimuli was different across conditions. Specifically, dogs remained oriented towards the presentation area for longer after being exposed to the slow condition, compared to the congruent or fast ones. Thus, experienced dogs were surprised by the delay, suggesting they had formed an expectation about the timing of reappearance. However, the same was not evident in the fast condition. One possibility to explain these results, is that time difference between the fast and the congruent condition (0.5 s) was not large enough to be detected by dogs, while the same was not true for the time difference between the congruent and the slow conditions (1 s). This seems unlikely, as durational discrimination in dogs, as well as in other species, is based on proportional differences, rather than absolute differences (Cliff et al. 2019; Heinrich et al. 2022; Vanmarle and Wynn 2006). Based on that, one would expect the opposite result, since the ratio between the timing of the congruent and fast condition (3.6) was larger than that between the slow and the congruent condition (2.4). An alternative explanation involves the possibility that, after the preliminary exposures, the dogs expected the ball to reappear and did not pay attention to the area of reappearance in advance, as also evident by their relatively high latency to orient to such area, compared to the actual timing of reappearance. In turn, this did not allow dogs to notice the premature reappearance. In other words, dogs were not surprised because the event they were expecting actually occurred, and not being already focussed on the area of reappearance, they could not detect the premature reappearance. Conversely, the slow condition resulted in dogs being surprised because the event they were expecting did not occur within the time frame they had learned through repeated exposures.

One further aspect that warrants discussion is about the nature of the information on which dogs generated their expectations. One possibility would be that dogs, by the preliminary exposures to the congruent condition, had habituated to the timing of the ball’s reappearance, and were hence surprised by the delayed timing of the slow condition, without implying the processing of information about the ball’s motion sensu* strictu*. This explanation is, however, unlikely: had experienced dogs habituated to the congruent condition through the two preliminary exposures, we should have observed a lower attention in the ball in the congruent trials, compared to unexperienced dogs, or a decrement in attention to the ball across trials, neither of which was the case. It, therefore, seems sensible to assume that dogs’ expectations about the ball’s reappearance were based on their ability to encode some aspects of its motion, rather than on a simple habituation to the timing of reappearance. How exactly different features of motion contribute to dogs’ ability to form these expectations remains to be clarified in future experiments.

## General discussion

In this study, we assessed whether dogs are able to expect the time and place of reappearance of a moving object with a partially occluded trajectory and the role of experience in such ability. Dogs that had not been previously exposed to the stimuli did not form an expectation about the time and place of the ball’s reappearance. To some extent, they were apparently able to track the movement of the ball when it disappeared, suggesting the involvement of a low-level (perceptual/behavioral) tracking mechanism. Conversely, dogs that were preliminary exposed to the congruent condition, were surprised when the ball stayed behind the occluder longer than expected, but showed no difference in latency to orient across conditions. Overall, the results suggest that experience allowed dogs to form an expectation about the ball’s movement, and to overcome the perceptual/behavioral automatism inherent in visual tracking.

Despite the apparent ability to predict the timing of the ball’s reappearance, experienced dogs did not show any anticipatory orientation towards the area after the barrier. Indeed, the latency by which these dogs oriented to the area of reappearance was not lower than 0—contrary to what would be expected if dogs were anticipating their orienting response—and similar to the one shown by naïve dogs in the congruent and the fast conditions. In contrast to these results, a study by Völter and colleagues (2020) found that through repeated exposures dogs’ gaze anticipated the movement of a frisbee thrown back and forth between two people, eventually fixing at the final location before the arrival of the frisbee itself. It is possible that the presence of a clear and visible stopping point (the person receiving the frisbee) facilitated the fixation of dogs’ anticipatory gaze on that point, whereas in the current experiment the ball did not stop at one location. The lack of specific end points might have led dogs to look elsewhere at its disappearance.

What remains unclear from the experiment by Völter and colleagues (2020) is on what basis experience led to dogs’ anticipatory looking. One parsimonious explanation would be that dogs learned a sequence of events, i.e., after one of the two individuals throws the frisbee, the other one will receive it, rather than learning and elaborating on some characteristic of the frisbee’s motion. A rapid acquisition about the frisbees’ behavior—reaching the receiver after leaving the sender—could be facilitated by the fact that dogs are likely exposed to similar situations (objects being thrown between one person and another) in real-life. Conversely, our stimuli entailed an abstract shape and motion (e.g., constant speed, lack of gravity) which could not resemble any real-life context. In this sense, the effect observed in our experienced dogs could only be the result of the two preliminary exposures to the stimuli and it suggests such brief experience was sufficient for dogs to learn some characteristics of the object’s motion.

A similar role of experience has been previously described in the ontogeny of motion prediction abilities in humans. Indeed, around six months of age, human infants are able to predict the reappearance of the object based on previous exposures and overcoming low-level tracking (Kochukhova and Gredebäck 2007). Interestingly, two presentations seem to be sufficient for human infants to form expectations and overcome the visual tracking mechanism (Kochukhova and Grebäck 2007), similarly to what we observed in the current experiment with dogs. Thus, it is possible that similar mechanisms guide the refinement linked to experience of motion prediction abilities in the two species.

## Conclusions

This study provides indications that dogs may resort to a perceptual/behavioral mechanisms that would allow them to maintain orientation towards a moving object, even when the latter temporarily disappears. As already observed in other species, the mechanism does not seem to convey an accurate ability, as the dog’s orientation is lagged compared to the actual spatiotemporal trajectory of the hidden object. In this sense, the study prompts a further exploration of the functional extents of the tracking mechanism, for instance to understand how much and how quickly the dog’s orientation slows down in relation to the characteristics of the object motion. Furthermore, an exploration of how different degrees or types of experience modulate the mechanism would also be a relevant extension of this research.

The study also indicates that in the lack of experience, dogs cannot form expectations about the spatiotemporal trajectory of objects. However, even a limited exposure seems to provide them with such ability. Nonetheless, we obtained supporting evidence of expectation only when the object reappeared with a sufficiently large delay compared to its correct timing, and our data cannot tell whether this was due to an insufficient sensitivity to differences in the timing of events, or to an inaccuracy in the expectation itself. These aspects should be clarified by further experiments. Moreover, considering the crucial role of experience highlighted by this study, further explorations on the role of experience are needed. For instance, it would be important to understand how different levels of exposure shape the ability to predict motion, as well as if and how experience with one specific type of motion would be generalized to motion with different features, such as changes in speed or direction. Moreover, an investigation of the ontogenesis of the ability to track and predict motion in dogs, possibly in comparative terms with well-known developmental trajectories of humans, is warranted. The ability to encode and use motion information in humans also changes with aging, in post-developmental age. The exploration of age-related changes in this ability in adult dogs is another potentially important area of extension of this research.

## Supplementary Information

Below is the link to the electronic supplementary material.

Supplementary file1 (PDF 82 KB)

Supplementary file2 (MP4 4222 KB)

Supplementary file3 (MP4 3723 KB)

Supplementary file4 (MP4 3262 KB)
