# Supplementary material for: Dogs’ ability to follow temporarily invisible moving objects: the ability to track and expect is shaped by experience
Source: Anim Cogn. 2022 Sep 27;26(2):523–35. doi: 10.1007/s10071-022-01695-5 (PMC9950174; doi:10.1007/s10071-022-01695-5)
Supplement: Supplementary file 1 — Supplementary file1 (PDF 82 KB) [file 10071_2022_1695_MOESM1_ESM.pdf]

Dogs' ability to follow temporarily invisible moving objects: ability to track and expect are shaped by experience.

Animal Cognition

Miina Lõoke[1], Orsolya Kanizsar[1], Cécile Guerineau[1], Paolo Mongillo[1]*, Lieta Marinelli[1]

[1]Department of Comparative Biomedicine and Food Science, University of Padua, Viale dell'Università 16, 35020, Legnaro (PD), Italy

Corresponding author: Paolo Mongillo, paolo.mongillo@unipd.it

Table 1. The age and breed of the dogs participating in Experiment 1.

Subject number	Breed	Age (y)
1	border collie	5
2	mixed	4
3	jack russell	3
4	mixed	3
5	australian shepherd	4
6	labrador retriever	3
7	golden retriever	4,5
8	border collie	4
9	smooth collie	3
10	shetland sheepdog	3
11	mixed	2
12	mixed	1,5
13	border collie	5
14	mixed	4,5
15	border collie	3

Table 2. The age and breed of the dogs participating in Experiment 2.

Subject number	Breed	Age (y)
1	Labrador retriver	3,5
2	mixed	4
3	mixed	9
4	german shepherd	2
5	mixed	3
6	mixed	1
7	mixed	10
8	boder collie	2
9	mixed	2
10	mixed	6
11	czechoslovakian wolfdog	6
12	mixed	4
13	cocker spaniel	6
14	siberian husky	1
15	mixed	11
16	german shepherd	2,5
17	mudi	5
18	galgo	1,5
19	boder collie	4,5
20	boder collie	3,5
21	bracco tedesco	5
22	cocker spaniel	6
23	labrador retriever	3,5
24	mixed	4
25	mixed	3
26	mixed	12
27	czechoslovakian wolfdog	4
28	australian shepherd	5
29	cocker spaniel	11
30	mixed	8
31	mixed	3
32	yorkshire	3
33	mixed	6
34	drahthaar	7
35	Labrador retriver	6
36	mixed	9
37	mixed	7
